# Predicting Sex From EEG: Validity and Generalizability of Deep-Learning-Based Interpretable Classifier

**DOI:** 10.3389/fnins.2020.589303

**Published:** 2020-10-27

**Authors:** Barbora Bučková, Martin Brunovský, Martin Bareš, Jaroslav Hlinka

**Affiliations:** ^1^Department of Cybernetics, Faculty of Electrical Engineering, Czech Technical University in Prague, Prague, Czechia; ^2^Department of Complex Systems, Institute of Computer Science of the Czech Academy of Sciences, Prague, Czechia; ^3^National Institute of Mental Health, Klecany, Czechia; ^4^Third Faculty of Medicine, Charles University, Prague, Czechia

**Keywords:** explainable artificial intelligence, EEG, sexual dimorsphism, classification, machine learning, major depressive disorder, biomarkers

## Abstract

Explainable artificial intelligence holds a great promise for neuroscience and plays an important role in the hypothesis generation process. We follow-up a recent machine learning-oriented study that constructed a deep convolutional neural network to automatically identify biological sex from EEG recordings in healthy individuals and highlighted the discriminative role of beta-band power. If generalizing, this finding would be relevant not only theoretically by pointing to some specific neurobiological sexual dimorphisms, but potentially also as a relevant confound in quantitative EEG diagnostic practice. To put this finding to test, we assess whether the automatic identification of biological sex generalizes to another dataset, particularly in the presence of a psychiatric disease, by testing the hypothesis of higher beta power in women compared to men on 134 patients suffering from Major Depressive Disorder. Moreover, we construct ROC curves and compare the performance of the classifiers in determining sex both before and after the antidepressant treatment. We replicate the observation of a significant difference in beta-band power between men and women, providing classification accuracy of nearly 77%. The difference was consistent across the majority of electrodes, however multivariate classification models did not generally improve the performance. Similar results were observed also after the antidepressant treatment (classification accuracy above 70%), further supporting the robustness of the initial finding.

## 1. Introduction

The use of machine learning (ML) in neuroscience has moved the field toward personalized medicine (Sejnowski et al., 2014). Indeed, the potential of advanced machine learning approaches, including deep learning algorithms, to construct complex predictive models is substantial and widely acknowledged, as is evident from the rapid growth of neuroscientific publications (Marblestone et al., 2016; Vogt, 2018; Glaser et al., 2019). The methods are applied to a broad spectrum of tasks, including but not limited to automatic alignment of neuroimages, segmentation and parcellation of the brain, improvement of the predictive performance of models, or benchmarking simple models by capturing complex nonlinear relationships between measured variables (Jenkinson and Smith, 2001; Fischl et al., 2004; Glaser et al., 2019). However, the rising complexity of the models considerably decreases our understanding of the architecture within, thus limiting the practical implementation of the findings. This particular constraint led to a concept of explainable neuroscience—shifting the focus purely from the quality of prediction to the data-driven hypothesis generation and ML inference (Samek et al., 2017; Vu et al., 2018). The framework outlines the use of ML in neuroscience, promising the potential to generate models unifying brain function and behavior, emphasizing interpretability and generalizability. An important part of this framework is the development of novel biomarkers (Woo et al., 2017; Langlotz et al., 2019). A biomarker is a characteristic that is objectively measured and evaluated as an indicator of normal biologic processes, pathogenic processes, or pharmacologic responses to a therapeutic intervention (De Gruttola et al., 2001). Biomarkers thus carry a considerable power to discriminate between groups they are derived from.

In 2018, van Putten et al. constructed a deep convolutional neural network to predict biological sex, analyzing 1,308 clinical EEG recordings of healthy patients, with the reported accuracy of 81% (van Putten et al., 2018). To provide more insight, on top of the classification itself, the authors performed a visualization and analysis of the filters of all six convolutional layers of the network, discovering that the algorithm classified preferably using the beta-band-derived features. In a subsequent step, they performed multivariate logistic regression using only the beta power from all channels and reached the accuracy of 70%. To the best of our knowledge, the report of Putten et al. remains the only successful attempt to automatically discriminate biological sex from clinical quality EEG data. As the poor reproducibility and generalizability of ML models have been denoted as the most significant pitfalls of machine learning (Sejnowski et al., 2014; Carlson et al., 2018), we decided to follow-up these findings by validation on an independent dataset. Moreover, if the beta activity is in fact a biomarker of biological sex, one may expect this difference to hold also in patients with neurological or psychiatric disorder. While of course, the prediction of biological sex from EEG is *per se* not a very efficient tool, if reproducible, it would point to potentially relevant biological sex-related differences in the processes generating the EEG signal, and understanding the existence of sex-related differences in EEG would be important for the practice of quantitative EEG assessment in both research and clinical practice.

We consequently decided to examine this finding on patients suffering from Major Depressive Disorder (MDD). MDD is a psychiatric condition that has been known for the alteration of the wake as well as sleep EEG patterns (Thibodeau et al., 2006; Olbrich et al., 2015). The alterations of EEG in MDD are comprehensively summarized by Olbrich et al. (2015), and include relatively inconsistent reports of the presence of alpha asymmetry, elevated absolute and relative alpha activity, and further changes in the slow-wave activity. While the EEG changes in depression could in principle affect the accuracy of sex classification, the reports of alteration of the beta activity are relatively sparse, although some authors indicated increased beta activity (Lieber and Prichep, 1988; Knott et al., 2001).

Broader research has been done on the identification of EEG activity alterations following the MDD treatment. There is evidence that antidepressant treatment changes the EEG patterns to an extent, making the outcome of the treatment partially predictable (Widge et al., 2018). As a change of pattern could negatively affect the performance of a biomarker, we decided to assess the performance of the beta-power independently for the EEG data acquired before and after antidepressant treatment.

In this article, we present an independent validation of the interpretable hypothesis formed by van Putten et al. (2018) based on their deep network analysis of EEG data. Moreover, we construct uni-variate and multivariate families of classifiers based on the EEG beta-band power to assess the discriminative power of beta-power in EEG as a sex biomarker in a sample of MDD patients. In order to control for the effect of treatment, we investigate the classification accuracy before and after the intervention.

## 2. Materials and Methods

### 2.1. Participants

A total of 144 participants with MDD were recruited. For details of the sample and recruitment criteria, see previous full reports of the clinical analysis (Bares et al., 2010, 2015a,b). The patients received 4 weeks of antidepressant treatment based on the decision of the psychiatrist. The distribution of treatments in the study was as follows: serotonin-norepinephrine reuptake inhibitors (53 patients); transcranial direct current stimulation (21 patients); repetitive transcranial magnetic stimulation (16 patients); selective serotonin reuptake inhibitors (16 patients); norepinephrine-dopamine reuptake inhibitors (11 patients); and other treatment (17 patients). Upon the initial preprocessing, we excluded 10 patients due to the technical difficulties with the EEG recordings, namely in six subjects the recordings were distorted and not readable, in four subjects, the recordings of three or more channels were silent. This resulted in the dataset consisting of 134 patients (93 women) with the mean age of 46 years (std = 11.7; min = 18; max = 65). Every participant was recorded twice, before and after the treatment. Prior to the study, the patients were informed about the design of the study, and each participant provided his/her informed consent. The study was approved by the ethical committee of the Prague Psychiatric Centre/National Institute of Mental Health. The design and all procedures adhered to the latest version of the Declaration of Helsinki and ICH/Good Clinical Practice guidelines.

### 2.2. EEG Recordings

We worked with 19 standard electrode positions that were common in all patients (while discarding from analysis any additional contacts available only in a subset of patients): Fp1, Fp2, F3, F4, C3, C4, P3, P4, O1, O2, F7, F8, T3, T4, T5, T6, Fz, Cz, and Pz. The EEG was recorded for 10 min in a sound attenuated room with subdued lighting, with patients in a semirecumbent position and eyes closed in a maximally alert state. During the recording, the alertness was controlled. If the patterns of drowsiness appeared in the EEG, the subjects were aroused by acoustic stimuli.

### 2.3. Data Processing

We adopted the EEGLab MATLAB toolbox for data processing. The cleaning process was inspired by the PREP pipeline (Delorme and Makeig, 2004; Bigdely-Shamlo et al., 2015). At first, the EEG was downsampled to 250 Hz. The initial and last 30 s of the recording were removed. Subsequently, the *clean rawdata* function was used. The function performs multiple operations: (1) Removes channels that have been flat for more than 5 s. (2) Applies a high-pass filter with 0.5 Hz cutoff frequency (transition width of the IIR filter: 0.25, 0.75). (3) Rejects the channels that are correlated with the neighboring channels less than a threshold (correlation threshold = 0.75). (4) Removes the bursts via Artifact Subspace Reconstruction—applies PCA decomposition to the channels in sliding window and rejects and reconstructs the components for which the standard deviation differs from the most representative part of the signal. The standard deviation threshold was set at 5 (Mullen et al., 2013; Plechawska-Wojcik et al., 2018). (5) Removes the unrepaired windows—a sliding window of 1 s and 66% overlap deletes the windows that contain more than four “bad channels.” The removed channels were interpolated using spherical interpolation. Finally, the data were re-referenced to average reference and a low-pass FIR filter was applied with a 40 Hz threshold. For each channel, the relative β band power was computed by dividing the power in the β frequency range [12–25 Hz] by the sum of the power in the four key frequency bands used in the original study (δ [0.5–4 Hz], θ [4–8 Hz], α [8–12 Hz], and β [12–25 Hz]).

### 2.4. Data Analysis

In order to test the presence of global beta power differences, we conducted a non-parametric Wilcoxon two-sample test on the mean relative beta-band power (i.e., averaged across all electrodes). We subsequently repeated the test to assess the differences in each individual electrode and corrected for multiple testing using Bonferroni correction. To assess the classification power of the mean relative beta power, we performed logistic regression on this feature and constructed the Receiver Operator Characteristic (ROC) curve. To rule out any potential bias due to the inequality of the sex ratio in our data (although plain logistic regression is generally robust again this), for all main analyses conducted in this study, we constructed additional models adhering to the following approach: (1) Randomly sample 40 males and 40 females. (2) Perform the logistic regression. (3) Construct the ROC curve. (4) Repeat the subsampling 100-times. As a result of this approach, we present the mean ROC curve over all the iterations.

As a further step toward potentially optimized classifier, multivariate logistic regression was applied in order to take advantage of the additional information that may have been present across the channels but could have been suppressed by using the average in the initial task. As in the univariate analysis, we constructed a full model including the relative beta power of all 19 channels and a separate averaged model for the sex-ratio balanced data. In order to evaluate and minimize the possibility of overfitting, the same procedure was repeated while applying a leave-one-out validation scheme.

We report the Area Under the Curve (AUC) and the highest overall accuracy across all thresholds that provided true positive rate above and false positive rate beneath 50%. The true and false positive rates are reported with respect to the prediction of the minor class in the data—men.

Moreover, for additional validation, the computations were performed twice, once for the data acquired before and once after the antidepressant treatment. All statistical analyses were run using Matlab (MATLAB, 2018).

## 3. Results

**Figure 1:**
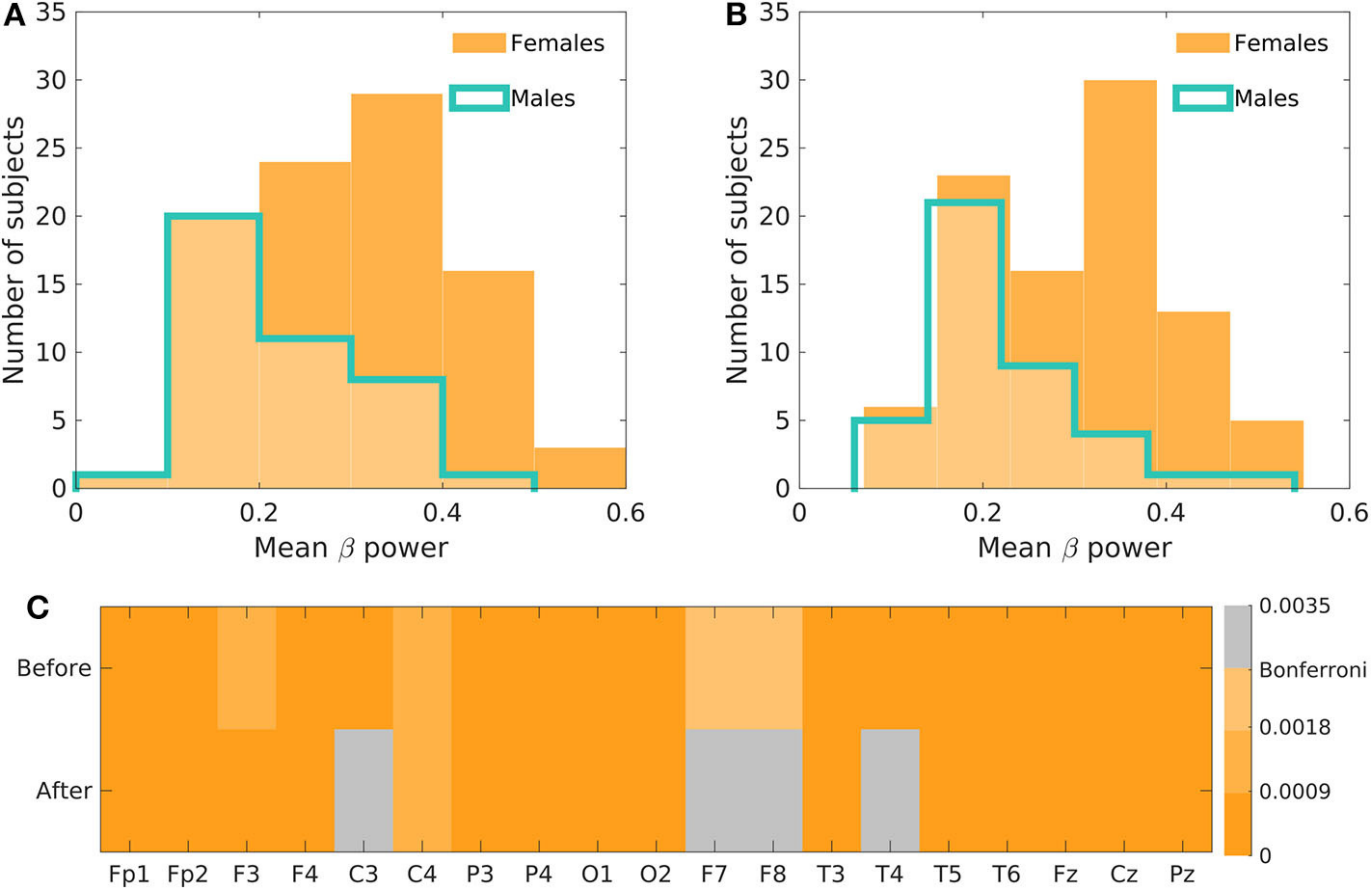
The initial assessment of the beta-power difference between men and women. **(A)** Depicts the histogram of the mean beta power in men and women before the antidepressant treatment and **(B)** after the treatment. **(C)** Shows the Bonferroni corrected *p*-values of the relative beta power differences across all electrodes before and after the treatment.

The initial test of the global differences between men and women in relative beta power showed a significantly higher relative beta power in women both before as well as after the antidepressant treatment (*p* < 0.001). The difference was apparent across all 19 electrodes when investigating the individual channels (Figure 1).

**Figure 2:**
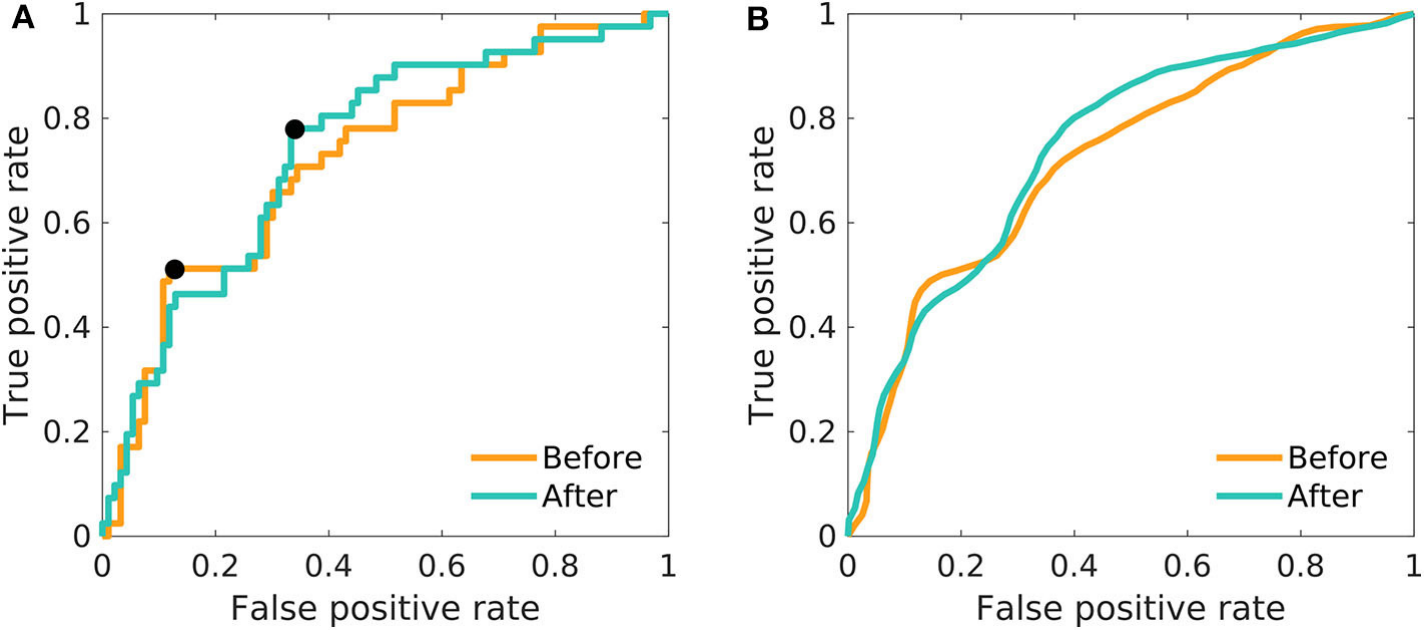
The ROC curves of one-dimensional logistic regression. Differences between men and women in the mean relative beta power; before as well as after the treatment. **(A)** The model was fitted on the whole dataset using the mean relative beta power. The black pointers indicate the position on the ROC curve, for which the overall accuracy is reported in Table 1. **(B)** The model was fitted 100-times on a random balanced subsample of 80 patients, and the resulting ROC-curves were averaged.

The use of one-dimensional logistic regression allowed powerful statistical evaluation of the full dataset without undergoing the risk of overfitting. The mean beta power feature generates the ROC curve with the AUC of 0.72 and 0.74 for the model before and after the treatment, respectively (Figure 2, Table 1). The highest accuracy (across thresholds for which the true positive rate was above and false positive rate beneath 50%) was 77 and 70% for the treatment before and after, respectively.

**Table 1:** Main results of the fitted models: In non-balanced models, the overall accuracy is reported as the highest accuracy reached (assessed across all thresholds providing true positive rate above 50% and false positive rate below 50%); for the position of the points on the ROC curves see Figures 2, 3.

	**Area under** **the curve**		**Overall accuracy for** **the chosen threshold**	
	**Before**	**After**	**Before (%)**	**After (%)**
Mean across the channels	0.7246	0.7425	76.87	70.15
Mean across the channels; balanced	0.7257	0.7257	69.14	72.01
All channels	0.8146	0.8652	77.61	79.85
All channels; balanced	0.8542	0.8941	79.09	83.45
All channels; leave-one-out	0.6420	0.7236	66.42	68.66
All channels; balanced; leave-one-out	0.5942	0.6481	61.47	66.55

*For balanced models, the average of overall accuracies across 100 subsamples is reported*.

**Figure 3:**
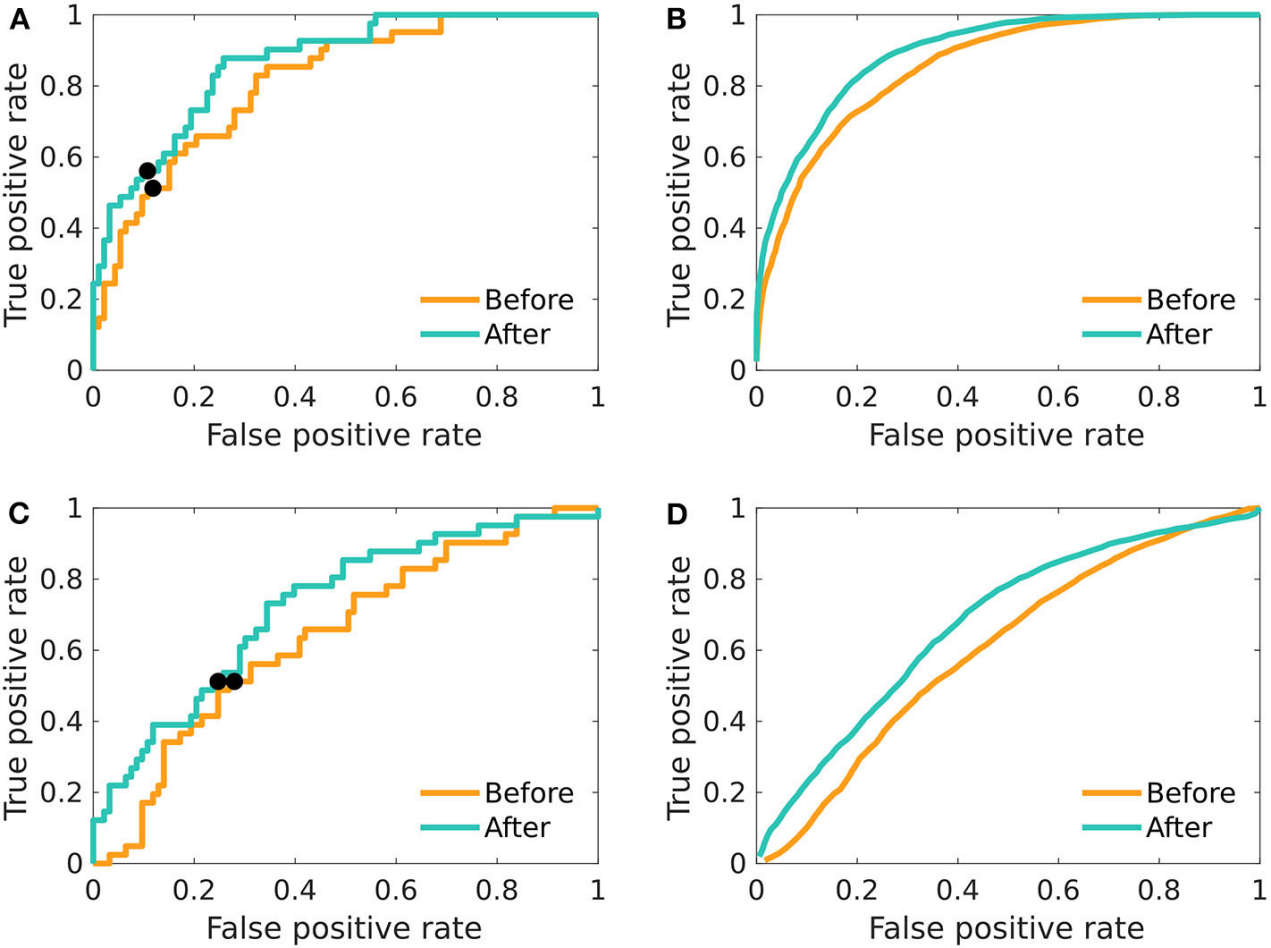
The ROC curves of multivariate logistic regression. Differences between men and women in relative beta power across electrodes; before as well as after the treatment. **(A)** The model was fitted on the whole dataset using the relative beta power across all 19 electrodes. **(B)** The model was fitted 100-times on a balanced random subset of 80 patients, and the resulting ROC-curves were averaged. **(C)** The model was fitted and evaluated using leave-one-out validation scheme. **(D)** The average of ROC created by using random sex-balanced subsets of the data and using leave-one-out scheme in fitting and evaluating the logistic regression. The black pointers in **(A,C)** indicate the position on the ROC curve, for which the overall accuracy is reported in the Table 1.

The adoption of multivariate logistic models did not provide higher accuracy. Furthermore, the resulting AUC and accuracy substantially decreased after applying the leave-one-out validation, showing that the concern of overfitting was justified. Figure 3A shows the overfitted models where all data were used in order to build the model. In both cases (before and after the treatment), the AUC is above 0.8. However, after applying the out-of-sample prediction (Figure 3C), the AUC decreased to 0.64 and 0.72 for the results before and after the treatment, respectively, which is inferior to the initial grand mean approach. The subsampling procedure showed that the results on the whole dataset are not systematically biased by the majority class (Figures 3B,D).

## 4. Discussion

Most authors agree that the ML approach to neuroscience has potential to bring substantial advances to the field (Sejnowski et al., 2014; Samek et al., 2017; Vogt, 2018; Langlotz et al., 2019). Nevertheless, it has been rightly pointed out that the problematic reproducibility and interpretability of results limits their practical use (Carlson et al., 2018). Indeed, searching within black boxes allows us to identify features with high classification or prediction potential, but our understanding of them is limited, unless they are used in simpler, hypothesis-driven models (Glaser et al., 2019). Such simpler models are more comprehensible and often more neuroscientifically valid (Woo et al., 2017). Although inferior in accuracy, they tend to be more robust, as they are less prone to overfitting due to the lower dimensionality (Whelan and Garavan, 2014). To ensure the validity of simpler models, we need to conduct confirmatory studies that would investigate the findings reported by the ML on independent data (Yahata et al., 2017).

In this study, we used the conclusions drawn by van Putten et al. (2018) from a deep learning study in a large sample of EEG data and decided to test for the relative beta-band power classification property with respect to biological sex. Working with the pre-defined hypothesis, we addressed two issues associated with the definition of biomarkers, namely testing the results on an independent dataset and examining the robustness of the biomarker even in the presence of a psychiatric disease.

In the statistical analysis, we focused solely on the confirmation of presence of the difference in the specific feature of *relative beta-band power* between men and women. This approach allowed us to minimize the amount of statistical testing, thus decreasing the probability of the occurrence of false positive findings. For this purpose, we have selected the logistic regression model as it is the model used in the original paper. The models containing only the mean relative beta-band power provided AUC above 0.72 and accuracy above 70% both before and after the treatment. Enhancing the models by using the relative beta-band powers from all individual channels did not significantly improve the diagnostic accuracy. In fact, due to the necessity to control for overfitting by out-of-sample testing, the resulting multivariate models gained complexity without significantly improving the predictions. Note that the AUC of the model using the mean beta power across channels is not prone to overfitting, as the only free parameter corresponds to the threshold that is varied across to provide the summary AUC measure.

Concerning the classification accuracy, only the maximum across a range of thresholds is reported, while in practice a specific working point is to be selected. However, the precise accuracy reached is meant to illustrate the strength of the differences rather than to aim toward devising a tool for diagnosing biological sex based on EEG. Rather, it suggests a substantial quantitative difference in the EEG signals between sexes that could point to some underlying differences in cognitive neurodynamics (see van Putten et al., 2018 for discussion of beta band differences to cognitive and emotional processing), or at the very least inform the EEG analysis practice of a potential confound of inter-subject analysis. Last but not least, it provides a proof of principle and a springboard for classification of clinically more relevant differences in EEG.

An interesting issue is that of using the multivariate or univariate model. In general, the accuracy reached by our one-dimensional model was consistent with the 70% accuracy reported by van Putten. Note that we have used the same definition of beta band as the authors of the original study (12–25 Hz), however, we decided to use relative spectral power that should be robust with respect to interindividual and inter-session variability in the signal amplitude. To assess the robustness of the result, we also computed the logistic regression model on the averaged absolute beta power, which resulted in just a slight decrease in the overall accuracy of the classifier: 68 and 66% for the conditions before and after the treatment, respectively.

In principle, the multivariate model can potentially more sensitively fit more complex, spatially dependent patterns of sexual dimorphism. On the other side, it is more prone to overfitting. The observed accuracy of the multivariate model is thus higher than for the univariate model, reaching up to 84% accuracy. On the other side, with a proper leave-one-out cross-validation, the accuracy falls to 61–69%. While in general the performance is thus comparable to that reported in the original work by van Putten et al. (2018), our results suggest that the use of simpler and more robust univariate model based on the single feature of mean relative beta power is more accurate.

Of course, steps could be taken to improve multivariate models' accuracy, such as using dimensionality reduction methods or changing the modeling strategy to algorithms more suitable for high-dimensional data. In the case of dimensionality reduction, a prior decision on the method and number of variables that ought to be present in the model is necessary. Furthermore, the method must be implemented correctly inside the cross-validation cycle to avoid double-dipping and prevent overoptimistic results (Maggipinto et al., 2017). To assess the role of the potential advantage of dimensionality reduction methods, we implemented a non-parametric Mann–Whitney test into leave-one-out cross-validation and compared maximum accuracy reached across the number of channels used in the model (the full results on all models are available in Supplementary Table 1). Overall, the condition of Bonferroni significance was not restrictive enough, due to the widespread differences between males and females (see Figure 1), which resulted in the accuracy inferior to the one-dimensional mean of all features. However, the reduction of the number of variables to four or less improved the performance and for the unbalanced dataset even marginally outperformed the one-dimensional mean model. Additionally, the support vector machines algorithm was used in order to compare the performance of full multivariate logistic regression models. Again, implemented in the cross-validation cycle, the support vector machines outperformed the mean logistic regression model on the data acquired after the treatment, but the classification accuracy on the data before the treatment was suboptimal, leading us to the conclusion that the logistic regression, used in the original study, was a suitable method to be used in our experiment setting. Of course, while our results provided additional support concerning the validity of the original hypothesis, further re-validation and generalization using independent datasets from both clinical groups as well as healthy subjects is warranted before widely utilized in practice.

We did not identify differences in the classification accuracy of the relative beta-band on data acquired before and after the subjects were given antidepressant treatment. In fact, the relative beta powers before and after the therapeutic intervention did not systematically change (paired *t*-test: *p* = 0.1997), and moreover they were significantly correlated across subjects both in the mean (*r* = 0.8824, *p* < 0.001) as well as for all channels (mean correlation = 0.7798, std. = 0.1018), supporting the existence of individually specific EEG signatures. Additionally, over a half of the patients that were incorrectly classified before the treatment, were also misclassified based on the data after the treatment (19 out of 31). Our observation of a negligible effect of the antidepressant treatment on beta power is in line with the current literature. Wade and Iosifescu (2016) described over 45 articles that derived quantitative EEG features in order to predict the depression treatment outcome. The most prevalent band-specific features were alpha-band activity, frontal theta activity, and theta cordance, whereas only one study reported decreased pre-frontal delta and beta cordance in non-responders (Arns et al., 2012), which indicates that this band is not affected by treatment and thus does not play a role in treatment outcome prediction. Furthermore, to rule out any possible confounding effect of the different treatments on the relative beta power, we have tested for differences between groups using two-way ANOVA (accounting for sex and group and controlled for age) and observed no effect of group, both prior (*p* = 0.53) and after (*p* = 0.62) the treatment.

To summarize, in agreement with the explainable neuroscience framework, we followed-up a previous deep-learning EEG study by testing for the presence of the inferred significant differences in the relative EEG beta-band power between men and women in an independent dataset. In order to test for the validity of this potential biomarker, we cautiously employed robust statistical approaches, which supported our hypothesis and provided classification accuracy of up to 77% in one-dimensional models. This illustrates the utility of explainable artificial intelligence approaches and independently supports a recent result concerning sexual dimorphism of EEG signals.

## Data Availability Statement

The raw data supporting the conclusions of this article will be made available by the authors, without undue reservation.

## Ethics Statement

The studies involving human participants were reviewed and approved by Ethical committee of the Prague Psychiatric Centre and Ethical committee of National Institute of Mental Health, Klecany, Czech Republic. The patients/participants provided their written informed consent to participate in this study.

## Author Contributions

MBa and MBr designed the study and acquired the data. BB and JH formulated the hypothesis and analysis design, analyzed, interpreted the data, and drafted the manuscript. All authors contributed to the manuscript revision and approved the final version for submission.

## Conflict of Interest

The authors declare that the research was conducted in the absence of any commercial or financial relationships that could be construed as a potential conflict of interest.
