# Supplementary material for: Predicting Sex From EEG: Validity and Generalizability of Deep-Learning-Based Interpretable Classifier
Source: Front Neurosci. 2020 Oct 27;14:589303. doi: 10.3389/fnins.2020.589303 (PMC7652844; doi:10.3389/fnins.2020.589303)
Supplement: Supplementary file 1 [file Data_Sheet_1.PDF]

# Supplementary Material

## 1 SUPPLEMENTARY TABLES

| Classifier | Number of features in the model | Measure | Before / After the treatment | All channels; leave-one-out | All channels; balanced; leave-one-out |
|---|---|---|---|---|---|
| SVM | full | AUC | before | 0.6279 | 0.6909 |
| | | | after | 0.7752 | 0.7394 |
| | | Accuracy | before | 55.97% | 73.13% |
| | | | after | 78.36% | 73.13% |
| LR | full | AUC | before | 0.6420 | 0.5942 |
| | | | after | 0.7236 | 0.6481 |
| | | Accuracy | before | 66.42% | 61.47% |
| | | | after | 68.66% | 66.55% |
| LR | Bonferroni | AUC | before | 0.6908 | 0.6747 |
| | | | after | 0.7144 | 0.6782 |
| | | Accuracy | before | 70.90% | 71.64% |
| | | | after | 72.39% | 70.15% |
| LR | 1 variable | AUC | before | 0.7367 | 0.7201 |
| | | | after | 0.7590 | 0.7234 |
| | | Accuracy | before | 73.88% | 73.13% |
| | | | after | 74.63% | 72.39% |
| LR | 2 variables | AUC | before | 0.7272 | 0.7022 |
| | | | after | 0.7634 | 0.7272 |
| | | Accuracy | before | 73.13% | 71.64% |
| | | | after | 76.12% | 71.64% |
| LR | 3 variables | AUC | before | 0.7438 | 0.7114 |
| | | | after | 0.7362 | 0.7330 |
| | | Accuracy | before | 73.88% | 70.90% |
| | | | after | 73.13% | 72.39% |
| LR | 4 variables | AUC | before | 0.7464 | 0.7006 |
| | | | after | 0.7149 | 0.7177 |
| | | Accuracy | before | 71.64% | 70.15% |
| | | | after | 70.15% | 69.40% |
| LR | 5 variables | AUC | before | 0.7152 | 0.6993 |
| | | | after | 0.7105 | 0.7111 |
| | | Accuracy | before | 71.64% | 73.13% |
| | | | after | 67.16% | 73.88% |
| LR | 10 variables | AUC | before | 0.6908 | 0.6642 |
| | | | after | 0.7144 | 0.6720 |
| | | Accuracy | before | 70.90% | 70.90% |
| | | | after | 72.39% | 70.15% |

**Table S1.** Additional classification results for the support vector machines (SVM) classifier using all variables, and for the logistic regression (LR) models with non-parametric Mann-Whitney test used to select the most significant variables. Due to the reasons mentioned in the article, the results are only reported for the models with cross validation cycle within.
